# Community-acquired mastitis due to *Mycobacterium abscessus*: a case report

**DOI:** 10.1186/1752-1947-3-130

**Published:** 2009-11-17

**Authors:** Maria Bruna Pasticci, Luigi Maria Lapalorcia, Giacomo Antonini, Antonella Mencacci, Rosanna Mazzolla, Franco Baldelli

**Affiliations:** 1 Infectious Disease Section, Department of Experimental Medicine and Biochemical Sciences, University of Perugia, Perugia, Italy; 2 Section Emergency Surgery, Hospital Santa Maria della Misericordia, Perugia, Italy; 3 Microbiology Section, Department of Experimental Medicine and Biochemical Sciences, University of Perugia, Perugia, Italy

## Abstract

**Introduction:**

*Mycobacterium abscessus *is a rapidly growing mycobacterium usually causing skin and soft tissue infections in immunocompetent patients following contaminated traumatic or surgical wounds or contaminated injected medications. Disseminated infections and pulmonary infections are usually reported in immunocompromised hosts.

**Case presentation:**

We describe a 54-year-old Caucasian woman with mastitis due to *M. abscessus*. A few days after clinical evidence of mastitis, the patient was started on broad-spectrum antibiotics. Subsequently, due to persistence of symptoms, a percutaneous breast biopsy was performed followed by surgical drainage. Initial cultures failed to grow micro-organisms and tissue histology showed chronic inflammatory reaction with giant cells. Several days after surgery, her symptoms recurred. Finally, *M. abscessus *breast infection was diagnosed and the patient was treated successfully.

**Conclusion:**

Rapidly growing mycobacteria need to be included in the differential diagnosis of patients with chronic mastitis having pus discharge and who do not respond to broad-spectrum antibiotics.

## Introduction

*Mycobacterium abscessus *is a rapidly growing mycobacterium [1,2] most commonly causing skin and soft tissue infections in immunocompetent patients following penetrating traumatic injuries and concomitant inoculation of *M. abscessus *into the host tissue [1,3]. Other settings of *M. abscessus *infections include post-surgical wound infection [3], eye surgery [4] and post-injection abscess [5]. Podiatric care [6] and body piercing [7] have also been associated with such infections. Clustered outbreaks or pseudo-outbreaks have resulted from contaminated ice, water, injected medications [8] or intra-mammary silicone. Disseminated infections and pulmonary infections are reported more often in immunocompromised patients and patients with cystic fibrosis [1,3,9].

We report the case of a patient with mastitis due to *M. abscessus *who had not undergone surgery nor suffered local trauma.

## Case presentation

A 54-year-old Italian, Caucasian woman with a history of autoimmune thyroiditis was seen at the surgical unit for pain, redness and swelling of the left breast which had developed over the preceding 72 hours. The patient had been on 10 mg prednisone for 1 month secondary to autoimmune hemolytic anemia. On physical examination, she was afebrile. Erythema and edema of both areolar and peri-areolar areas of the left breast were present with dimensions of about 9 ×6 cm and with homolateral tender lymph node enlargement. Acute bacterial mastitis was the initial diagnosis and cefotaxime 2 g/day was prescribed for the first 2 weeks. At this point, due to persistence of symptoms, breast ultrasound, mammography, fine needle aspiration, and tru-cut needle biopsies were performed. Histopathology results were consistent with a chronic inflammatory reaction. Subsequently, a draining sinus developed and a pus sample was sterile.

**Figure 1:**
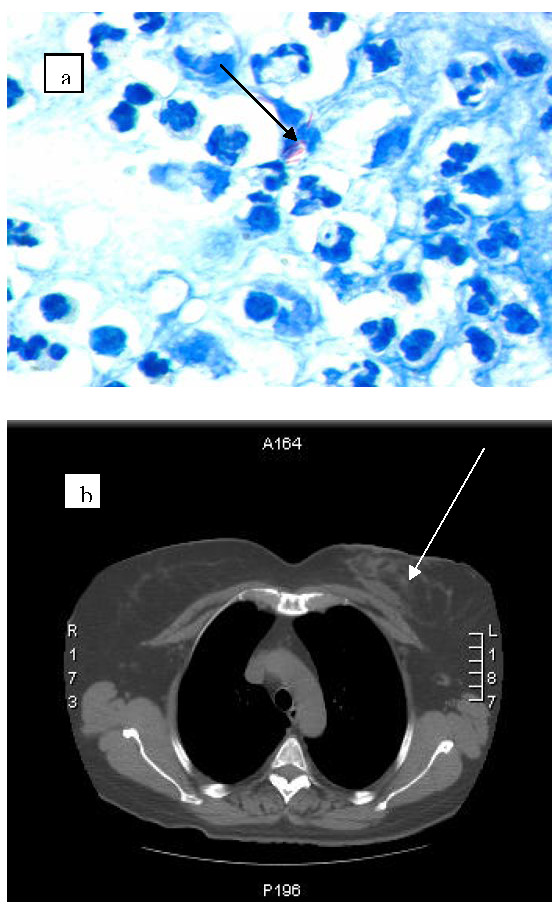
**(A) Acid fast bacilli in pus (arrow) on Zhiel-Neelsen staining; (B) Chest CT scan showing a soft tissue abscess 4.5 × 3 cm (arrow) with a cutaneous fistula**.

One week later, another swab grew both *Pseudomonas luteola *and *Staphylococcus epidermidis *prompting us to perform a second needle aspirate. Cefotaxime 1 g TID and oral ciprofloxacin 500 mg BID were prescribed for a total of 3 weeks. During this period, mild improvement was seen but as the fistula closed her symptoms relapsed so it was decided to perform surgical excision of the abscess. At surgery, galactophora ducts were found to be filled with purulent material. Also a mass of 9.5 × 7 × 5 cm, with cavitation and purulent material, was excised. Once again, histopathology reports showed a chronic inflammatory reaction with giant cells. About 1 week after surgery, the inflammation reappeared and a third needle aspirate was performed. This time, *M. abscessus *was identified and confirmed in two subsequent cultures [1,2]. Acid-fast bacilli (AFB) were also seen in the pus after Zhiel-Neelsen staining (Figure 1A). At this point, the patient was transferred to the infectious diseases service.

On admission, the patient had an area of inflammation of about 6 × 7 × 4 cm in the external quadrants of her left breast. A few days later, a fistula formed and microbiological tests confirmed the presence of *M. abscessus*.

The isolated strains were identified by the reverse hybridization method (INNO-LiPA, Belgium) and confirmed by genetic sequencing [1,2]. Antimycobacterial susceptibility tests were performed in microdilution broth (MHB, BioMérieux, France) [10]. *M. abscessus *results were as follows: susceptible: clarithromycin (MIC 0.125 mg/L) and amikacin (MIC 2 mg/L); intermediate susceptible: cefoxitin (MIC 16 mg/L) and imipenem (MIC 8 mg/L); resistant: ciprofloxacin (MIC 8 mg/L), doxycycline (MIC 8 mg/L), co-trimoxazole (MIC >64 mg/L) and linezolid (MIC 32 mg/L). Abnormal laboratory test results are listed in Table 1. HIV serology was negative, CD4+ lymphocytes were normal and blood cultures were negative for AFB. Therapy consisting of clarithromycin 500 mg BID, imipenem 1 g BID and amikacin 1 g/day was started. The latter was discontinued after 2 weeks due to possible vestibular side effects. Prednisone was also discontinued without recurrence of hemolysis. A chest CT scan confirmed left breast soft tissue and glandular involvement (Figure 1B). Thirty-two days after admission, the inflammation was considerably reduced and the patient was discharged on clarithromycin 500 mg twice a day. Only 1 week later, the patient had to be readmitted because of recurrence. An ultrasound scan showed a hyperechogenic area with hypoechogenicity and aspiration was performed. This time, AFBs were not identified in the pus and the cultures were negative. Imipenem was restarted with clarithromycin for a further 3 weeks. Thereafter, clarithromycin alone was prescribed for another 10 weeks. Eighteen months after treatment discontinuation, the patient remains disease-free.

**Table 1:** Clinical, laboratory findings and therapy

Date	Clinical signs	Hb	RBC × 10^9^	ESR (mm/h)	CRP (mg/dL)	α_2_-globulin	Z-N		Therapy
16 December 2006	Edema, erythema (5 cm)	10.5	2.82	46	1.6	13.6	POS	POS	I+C+A (32 days)
18 January 2007	Normal	11.5	3.38	18	0.2	12.5	ND	ND	C (9 days)
27 January 2007	Edema, erythema (3 cm)	11.9	3.44	23	0.9	12.3	NEG	NEG	I+C (21 days)
15 February 2007	Normal	12.3	4.76	20	0.5	11.8	ND	ND	C (75 days)

I, imipenem; C, clarithromycin; A, amikacin; Hb, hemoglobin; RBC, red blood cell count; ESR, erythrocyte sedimentation rate; CRP, C-reactive protein Z-N, Zhiel-Neelsen staining; ND, not done.

## Discussion

Most *M. abscessus *infections in immunocompetent patients are caused by either post-traumatic or post-surgical skin and soft tissue inoculation of bacteria [1,3] and some have nosocomial origins [3]. Our patient had no soil or gardening exposure, and did not report any other exposures predisposing to *M. abscessus *infection. Moreover, it is most likely that the infection was community-acquired and its source cannot be established. The low dose of steroids administered one month before *M. abscessus *mastitis may have produced some negative immunomodulatory effects predisposing her to infection.

Etiological diagnosis did not occur immediately. Routine bacterial cultures would not be expected to grow mycobacterium, even rapidly growing species.

*M. abscessus *is resistant to several antibiotics [3,10]. Also, this isolate was only susceptible to clarithromycin and amikacin, while cefoxitin and imipenem had intermediate activity. Our patient received cefotaxime, which had not been tested *in vitro*, and ciprofloxacin which was resistant. In the end, only the combination of clarithromycin, imipenem and amikacin was truly effective. Side effects included vestibular toxicity and were very likely due to amikacin. This antimicrobial was, therefore, discontinued after 2 weeks and vestibular symptoms regressed. Imipenem and clarithromycin were continued for a total of 4 and a half months [3]. Stains and cultures were negative after the tenth week of treatment.

## Conclusion

*M. abscessus *is a rare cause of skin and soft tissue infection and should be considered in the differential diagnosis of patients who do not respond to standard antibacterial therapy.

## Abbreviations

AFB: acid-fast bacilli.

## Consent

Written informed consent was obtained from the patient for publication of this case report and accompanying image. A copy of the written consent is available for review by the Editor-in Chief of this journal.

## Competing interests

The authors declare that they have no competing interests.

## Authors' contributions

MBP was involved in the diagnosis and treatment of the patient and revised the manuscript critically for content. GA was the main surgeon and was involved in revising the draft. LML was involved in drafting the manuscript. AM and RM carried out the microbiological diagnosis and were involved in revising the manuscript. FB revised the final draft of the manuscript.
